# Turning away from danger

**DOI:** 10.7554/eLife.59910

**Published:** 2020-07-21

**Authors:** Jun Liu, Monika Scholz

**Affiliations:** 1 Max Planck Research Group Neural Information Flow, Center of Advanced European Studies and ResearchBonnGermany

**Keywords:** motor sequence generation, feedforward excitation, winner-takes-all, escape response, mutual inhibition, *C. elegans*

## Abstract

The flexible escape behavior exhibited by *C. elegans* in response to threats relies on a combination of feedback and feedforward circuits.

**Related research article** Wang Y, Zhang X, Xin Q, Hung W, Florman J, Huo J, Xu T, Xie Y, Alkema MJ, Zhen M, Wen Q. 2020. Flexible motor sequence generation during stereotyped escape responses. *eLife* **9**:e56942. doi: 10.7554/eLife.56942

Escaping from predators is an ancient problem. In 2007, for example, researchers reported evidence for a small worm trying to escape from a carnivorous fungus in a piece of amber that was about 100 million years old (Schmidt et al., 2007; Figure 1A). In response to a threat, an animal must translate sensory information into a fast getaway, and many animals use stereotyped responses (this is, instinctive behaviors that are not learned). Examples include the tail-flip escape in crayfish (Edwards et al., 1999), flight initiation in fruit flies (Card, 2012) and the withdrawal response in mollusks (Katz, 1998). Studying these responses has provided useful insights into the neuronal processes involved in fast, pre-programmed movements.

**Figure 1.**
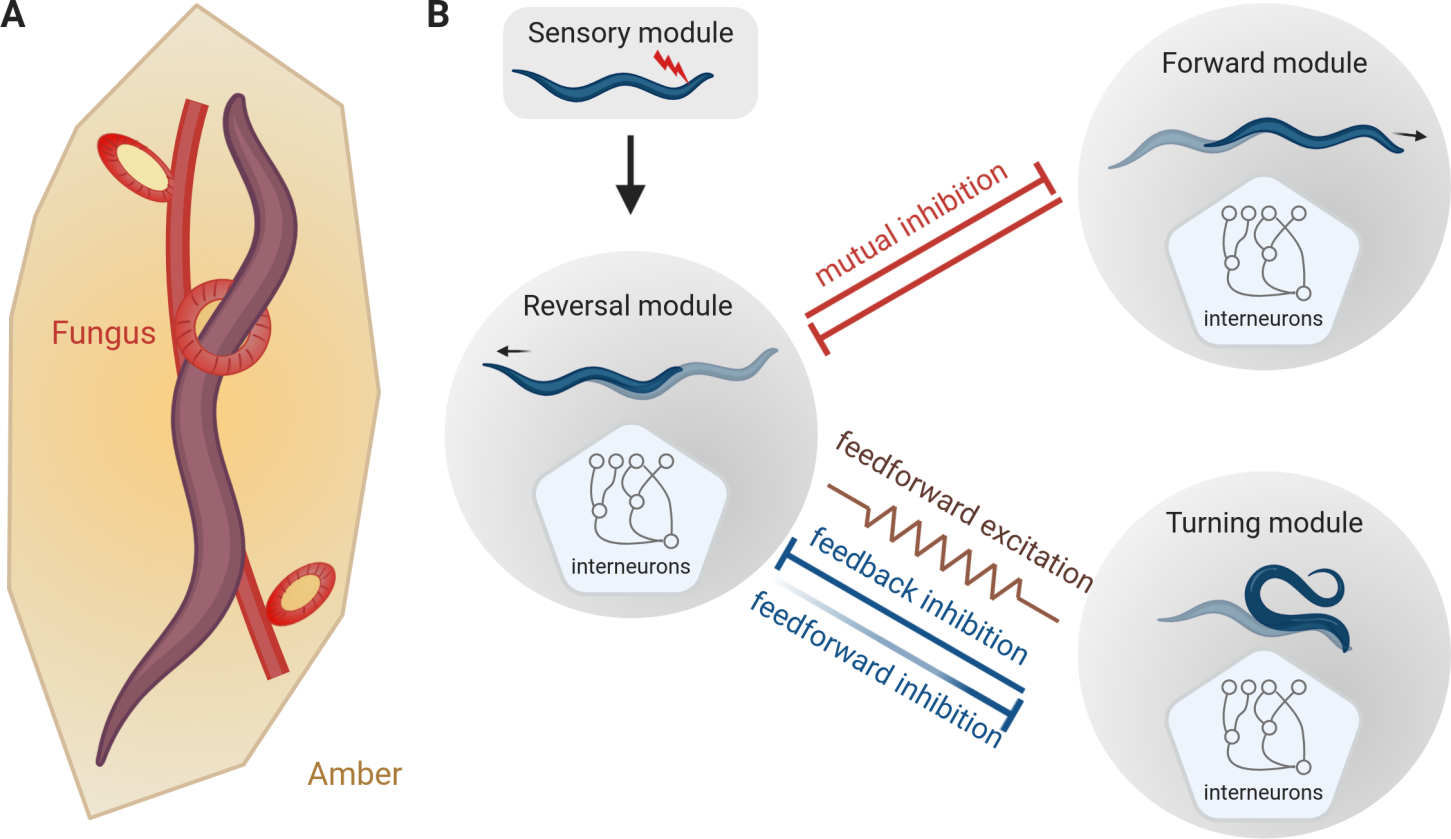
The escape response of *C. elegans*. (**A**) Roundworms have been trying to escape from predators for millions of years. This schematic, based on analysis of a piece of amber that is about 100 million years old, shows a worm being trapped by a carnivorous fungus (Schmidt et al., 2007). (**B**) When a worm encounters an unpleasant stimulus (red flash, top), it escapes by backing away. Subsequently it starts to either move forward again (top right) or to turn and move in a new direction (bottom right). Interactions between the neural modules that control these three types of motion result in flexible outcomes. Mutual inhibition (red flat-ended arrows) between the reversal module and the forward module explains why the rate of reverse-forward transitions does not change with the length of the reversal. The relationship between the reversal module and the turning module is more complex as it involves both feedforward and feedback inhibitions, (blue flat-ended arrows) and feedforward excitation (brown zig-zag line). Moreover, the work of Wang et al. suggests that the feedforward inhibition from the reversal module to the turning module weakens with time (shown here with fading), thereby suggesting how longer reversals are more likely to be followed by turns.

However, these stereotyped responses can be exploited by a clever predator if they become too predictable. How do animals strike a balance between the advantages of fast stereotyped movements and the need to avoid being too predictable? Now, in eLife, Quan Wen (University of Science and Technology of China and Chinese Academy of Science) and colleagues in China, Canada and the United States – including Yuan Wang, Xiaoqian Zhang and Qi Xin as joint first authors – report the results of experiments on the roundworm *Caenorhabditis elegans* that shed light on this question in nematodes (Wang et al., 2020).

Upon encountering danger, *C. elegans* will first move away, a behavior called reversal (Pirri and Alkema, 2012). After the reversal, a worm can either resume movement in the original direction (reversal-forward), or it can bend its body into an omega shape, with its head touching its tail, and change direction (reversal-turn). Wang et al. used optogenetic techniques to 'threaten' worms by simulating an unpleasant touch sensation, and observed how they responded (red flash in Figure 1B). Similar to prior observations, the experiments showed that the longer the reversal, the more likely it was to be followed by an omega turn (Zhao et al., 2003; Gray et al., 2005). However, the rate of resuming forward motion was independent of the length of the reversal. This observation suggests that worms compute their escape response in a way that allows them to remain flexible.

Interneurons are sandwiched between the sensory neurons that interpret the environment, and the motor neurons that drive movement and are important for neural computation. Previous research showed that some interneurons can regulate the frequencies of reversals or turns in *C. elegans* (Gray et al., 2005). But how do they modulate the ratio of reverse-forward versus reverse-turn transitions? Since the rate of reverse-forward transitions does not change with the length of the reversal, Wang et al. suggested a simple mutual inhibition model in which the module that controls reverse motion blocks the module that controls forward motion, and vice versa (red flat-ended arrows in Figure 1B). This mutual inhibition appears to happen through chemical synapses between interneurons in the two modules (Li et al., 2014; Wang et al., 2020).

Wang et al. then explored if the escape response of *C. elegans* involved feedforward excitation, a common type of neural circuit in which each neuron in a chain excites the next one. Circuits of this type are responsible for generating various forms of stereotyped behavior, such as bird song (in which each syllable is precisely timed: Pehlevan et al., 2018). Indeed, some of the interneurons in the reversal module showed feedforward excitation into a group of interneurons in the turning module. Gap junctions (which serve as regulated gates for communications between cells) are required for the feedforward activation, since worms with defective gap junctions performed fewer escapes that ended with a turn (brown gap junction, indicated by a zig-zag line, in Figure 1B).

However, if feedforward excitation was the sole contributor to reverse-turn transitions, altering the activity of downstream neurons should not affect the neurons earlier in the chain. In contrast, inhibiting the turning module caused prolonged reversals, suggesting that the turning module inhibits the reversal module, helping to end the reversal and start the turn. Conversely, for worms that have been genetically modified to prevent the secretion of inhibitory neurotransmitters in the reversal module, the transition from reversal to turning is much quicker, indicating that the reversal module inhibits the turning module (blue flat-ended arrows in Figure 1B). This means that in addition to feedforward excitation, the reverse-turn transition is also controlled by both feedforward and feedback inhibitions.

Wang et al. then built a model of interneurons to reproduce the experimental rates of reverse-forward and reverse-turn transitions. This model revealed that feedforward excitation and feedback inhibition were not sufficient to obtain the experimental reverse-turn transition rate. Weakening the inhibition of turning from the reversal module over time resolved this issue, which could explain why long reversals are more often followed by omega turns (fading blue flat-ended arrow in Figure 1B). At the molecular level this could be explained by the neurotransmitter responsible for the inhibition being depleted over time.

Collectively, the feedforward and feedback inhibitions between the reversal and turning modules lead to a winner-takes-all strategy: the more active module inhibits the other module and stays active. It remains to be seen what establishes the overall balance between escapes that end with forward movement and those that end with a turn, and how this may depend on each worm’s internal state and its history (Zhao et al., 2003). One could imagine that, after a particularly harmful stimulus, a long reversal and a reorientation are chosen to avoid returning to the same spot.
